# Subjective health complaints in early adolescence reflect stress: A study among adolescents in Western Sweden

**DOI:** 10.1177/14034948211008555

**Published:** 2021-04-16

**Authors:** Maria Corell, Peter Friberg, Petra Löfstedt, Max Petzold, Yun Chen

**Affiliations:** School of Public Health and Community Medicine, Institute of Medicine, Gothenburg University, Gothenburg, Sweden

**Keywords:** Subjective health complaints, stress, adolescents, Sweden, socioeconomic conditions

## Abstract

*Aims:* Mental health problems are common among Swedish adolescents and are sometimes referred to as ‘stress-related’. The overall aim of this study is to do an analysis of subjective health complaints (SHCs) and perceived general stress among adolescents in Sweden, both their prevalence and association, by gender, migration background, family structure and socioeconomic conditions. *Methods:* Data from the baseline (comprising 2283 adolescents aged 13) of the STudy of Adolescence Resilience and Stress (STARS) study in Västra Götaland in Sweden were used. SHCs were measured by the Psychosomatic Problems Scale (PSP-scale) and self-reported stress was measured by Cohen’s Perceived Stress Scale (PSS-10). Socioeconomic conditions were measured with the Family Affluence Scale (FAS) and the MacArthur Scale of Subjective Social Status (SSS). Statistical analyses included Student’s *t*-tests and ANOVAs of means, linear and logistic regression analyses and Pearson’s correlations. *Results:* Social inequalities in both SHCs and self-reported stress were found; levels were higher among girls, adolescents living with one parent or in families with less favourable socioeconomic conditions. Self-reported stress and SHCs were found to be strongly correlated (*r*=0.70). Correlations with self-reported stress were stronger for psychological complaints (*r*=0.71) than for somatic complaints (*r*=0.52). Correlations did not vary with socioeconomic conditions of the family. *Conclusions:* SHCs do reflect general stress among adolescents, and it is appropriate to address the complaints as ‘stress-related’. Measures to improve adolescents’ mental health by reducing levels of SHCs should pay special attention to stressors in adolescents’ daily lives and strengthening adolescent’s coping resources and strategies.

## Background

Self-reported mental health problems are common during adolescence and the problems have increased in many countries, including Sweden [1, 2]. Mental health problems during adolescence may impair everyday functioning such as school performance [3] and may persist into adulthood, affecting chances of education, employment and health [2, 4].

Subjective health complaints (SHCs ) are reported more frequently among girls than boys, older than younger adolescents and the gender difference in reported health complaints increases with age [5-7]. Results regarding Sweden are confirmed in other Swedish self-reports to children and adolescents [8, 9].

In most European countries, SHCs are reported more frequently among adolescents in families with low objective socioeconomic status, measured with the Family Affluence Scale (FAS) [6, 10]. In Sweden, subjective socioeconomic status has been shown to be more important for adolescents’ health than objective socioeconomic status [11, 12].

The last decades’ increase of mental ill health, both mental health problems [13] and mental disorders [14], among young people in Sweden is a public health problem with potentially serious consequences for both individuals and society [3, 4]. Several attempts to identify the main causes of the increase in mental ill health have taken place, and changes in the school system and the labour market, along with increased individualization, have been suggested as potential causes [15]. In Sweden, as well as in other countries, there has been a discussion whether or not the increase is real or reflects changes in reporting [1, 2] and if there has been a medicalization of normal, everyday problems [2]. There is therefore a need for more in-depth knowledge of its causes in order to both prevent and treat mental ill health.

While the prevalence of SHCs and their social determinants among children and adolescents are well monitored in many countries, including Sweden, less is known about the underlying mechanisms of SHCs. They are sometimes referred to as ‘stress-related’ [10, 16, 17], but few studies have actually explored the links between perceived general stress and SHCs among children and adolescents.

The Stress and Coping Theory defines stress as a relationship between the person and the environment. The theory consists of four elements: an internal or external stressor, an appraisal of the stressor, a coping strategy and a stress reaction (psychological and physical) [18]. The theory states that an individual’s appraisal and coping of stressors are influenced by individual and environmental factors. Pearlin’s Stress Process model has a similar approach but emphasizes the importance of the social conditions in which people live for their health [19]. Thus, poor social conditions may both act as chronic stressors and negatively influence individual’s coping resources and strategies.

**Figure 1.**
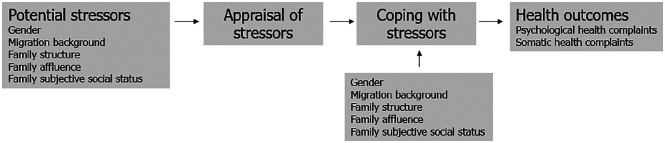
The potential links between social conditions, stress and subjective health complaints.

In this study, and in line with the Stress and Coping Theory, we hypothesize that stressors in adolescents’ daily lives may result in SHCs if they are appraised as stressful and met by insufficient coping responses. In accordance with the Stress Process Model, we also hypothesize that gender, along with social conditions, such as migration background, family structure and socioeconomic conditions, may both act as stressors in themselves and influence adolescents’ coping processes and affect their health (see Figure 1).

On the one hand, previous studies have shown that self-reported stress is in fact related to both somatic and psychological complaints among children and adolescents [20-23]. On the other hand, there is a lack of studies using composite measures of both stress and SHCs and also take social conditions into account when looking at the associations. There is therefore a need to further explore the association between perceived stress and SHCs, focusing on social inequalities.

### Aims

The overall aim of this study is to do an analysis of SHCs and perceived general stress among adolescents in Sweden and analyse their prevalence and association. The research questions are:

1. Do levels of SHCs and perceived general stress among adolescents vary with gender, migration background, family structure and socioeconomic conditions?
2. Is there an association between perceived general stress and SHCs among adolescents, and if so, does the association vary with gender, migration background, family structure, and socioeconomic conditions?

## Methods

### Data

Data from the baseline (2015−2019) of the STudy of Adolescence Resilience and Stress (STARS), comprising 2283 children from 54 schools in 16 municipalities in the region of Västra Götaland, were used. Schools were selected from areas with various socioeconomic contexts. With consent from the principals, researchers visited 7th grade classes to inform students and their teachers about the study. Next, information letters were sent to students and their parents or guardians. Written consent from both students and parents (or guardians) was obtained before participation. The response rate was 45 per cent. Ethical approval was obtained by the Regional Ethics Board in Gothenburg (Dnr 578-15).

### Variables

SHCs were measured by the Psychosomatic Problems (PSP)-scale which is intended to measure psychosomatic problems among schoolchildren and adolescents in general populations. The scale has been validated using the Rasch model on data for Swedish adolescents aged 15−16 years: the scale shows high reliability and works invariantly over time and between genders [24].

The PSP-scale is introduced with ‘Your well-being and your health (if you think of the last 6 months. . .) Have you had difficulties concentrating, difficulties in getting to sleep, headache, stomach ache, felt tense, had a bad appetite, felt sad or felt dizzy?’. The response options range from *never* to *always*, coded from 0 (never) to 4 (always) in this study, resulting in sum scores 0−32 for total complaints, 0−12 for somatic complaints and 0−20 for psychological complaints. The cut-off ‘at least two health complaints, often or always’ was used in the logistic regression analysis. Students with data for at least seven health complaints were included (*n* = 2275*)* in the analyses. Missing items were replaced by the intrapersonal mean of PSP-scale before a total score was calculated. In our sample, the Cronbach’s alpha was α = 0.837.

*Self-reported stress* was measured by Cohen’s Perceived Stress Scale (PSS-10). Items were designed to measure how unpredictable, uncontrollable and overloaded respondents find their lives. The scale also includes one item about current levels of experienced nervousness and stress. The PSS-scale shows adequate internal and test–retest reliability [25]. In our sample, the Cronbach’s alpha was α = 0.812.

The questions concern the respondent’s feelings and thoughts during the last month. The response options range from *never* to *very often*. For questions 1, 2, 3, 6, 9 and 10 the response options were coded from 0 (never) to 4 (very often), while response options for questions 4, 5, 7 and 8 were reversely coded. Only students who answered at least nine questions were included (*n* = 2276). Missing items were replaced by the intrapersonal mean of PSS-scale before a total score was calculated. The higher the values (0−40), the higher the stress. The quartile of students with the highest levels of stress was analyzed in the logistic regression analysis.

*Migration background* was derived from three questions about the student’s, the mother’s and the father’s country of birth. In this study, and in line with current guidelines, students who were born in Sweden, and have at least one Swedish-born parent, were coded as Swedish background. Students who were born abroad or have both parents born abroad were coded as foreign background.

*Family structure* was addressed by one question. We divided students into students who live with both parents, in joint custody or with one parent (always or mostly). Students who live without their parents (*n* = 16) were coded as missing.

*Family affluence* was measured through six questions regarding car ownership, own bedroom, computer ownership, number of bathrooms, dishwasher and number of holidays abroad. Sum scores (0−13) were calculated. The scale was developed by the HBSC network and has been validated several times (most recently by Torsheim et al. [26]). According to HBSC guidelines [27, 28], as affluence varies greatly across countries, a ridit-based division of students into quintiles should be made for each country. The division of students should be 20/60/20 per cent, corresponding to low/medium/high FAS. In this study, the following cut-offs were used; low (0−8), medium (9−11) and high (12−13) family affluence (27/59/14 per cent).

*Subjective social status (SSS)* was measured by the MacArthur Scale of Subjective Social Status – Youth Version [29]. Students were asked to mark the rung that best represents where their family would be on a ladder picturing how Swedish society is set up. There are no recommended cut-offs for the scale; hence, we grouped the students into three groups: students who marked rung 1−4 were grouped into low, rung 5−6 into medium and rung 7−10 into high subjective social status.

### Analysis and statistics

As both SHCs and perceived stress were normally distributed, Student *t*-test and ANOVAs with Bonferroni correction were performed to determine if there were statistically significant differences in mean values between groups. To study differences in multiple health complaints and high levels of stress between groups, logistic regression analyses were performed. To study the association between self-reported stress and SHCs, Pearson’s *r* values were calculated, using the sum scores of SHCs and self-reported stress. Fisher’s r-to-z transformation was used to determine if there were statistically significant differences in correlations between subgroups. Also, multiple linear correlations were performed to investigate possible confounders.

IBM SPSS 26 was used. A two-sided *p*⩽0.05 was considered as statistically significant.

## Results

### The prevalence of SHCs and self-reported stress

The prevalence of SHCs and self-reported stress are shown in Table I. Results regarding SHCs show that mean values were significantly higher among girls than boys. Levels of total SHCs were similar among adolescents with Swedish and foreign background, although levels of psychological complaints were higher among the former and somatic complaints higher among the latter. Levels of complaints varied with family structure, where adolescents living with one parent had higher mean values of SHCs than those living with both parents. Psychological complaints were higher among adolescents in joint custody and those with one parent than those living with both parents. SHCs, both total and the two subscales, also varied with the family’s socioeconomic conditions; adolescents in families with low family affluence had higher levels than those in high affluence. Adolescents who perceived their families’ SSS as low or medium had higher levels of SHCs than those who perceived their families’ SSS as high.

**Table I.** Distribution of students, subjective health complaints (SHCs) and self-reported stress among 13-year-olds, after gender, migration background, family structure and socioeconomic conditions, STARS 2015-2019.

	Students		Total complaints (0–32)			Somatic complaints (0–12)			Psychological complaints (0–20)			Stress (0–40)		
	*n*	%	Mean	*p*	*n*	Mean	*p*	*n*	Mean	*p*	*n*	Mean	*p*	*n*
*Gender*														
Boys (ref)	*1015*	44.5	9.9		*1011*	3.6		*1012*	6.3		*1011*	13.9		*1010*
Girls	*1268*	55.5	**13.1**	*p⩽*0.001	*1266*	**4.6**	*p⩽*0.001	*1266*	**8.4**	*p⩽*0.001	*1266*	**16.9**	*p⩽*0.001	*1266*
Missing	*0*	0.0												
*Migration background*														
Swedish background (ref)	*1793*	78.5	11.7		*1793*	4.1		*1793*	7.6		*1793*	15.4		*1793*
Foreign background	*478*	20.9	11.6	0.766	*477*	**4.4**	0.016	*478*	**7.2**	0.039	*477*	**16.1**	0.020	*476*
Missing	*12*	0.5												
*Family structure*														
Both parents (ref)	*1691*	74.1	11.3		*1688*	4.0		*1689*	7.3		*1688*	15.2		*1687*
Joint custody	*254*	11.1	12.0	0.235	*253*	4.1	1.000	*253*	**7.9**	0.043	*253*	15.7	0.631	*253*
One parent	*321*	14.1	**13.1**	*p⩽*0.001	*320*	**4.6**	*p⩽*0.001	*320*	**8.5**	*p⩽*0.001	*320*	**17.3**	*p⩽*0.001	*320*
Missing	*17*	0.7												
*Family affluence*														
Low	*619*	27.1	**12.3**	0.013	*617*	**4.5**	0.021	*618*	**7.8**	0.036	*617*	**16.6**	*p⩽*0.001	*617*
Medium	*1343*	58.8	11.5	1.000	*1341*	4.0	1.000	*1341*	7.4	0.904	*1341*	15.2	1.000	*1340*
High (ref)	*320*	14.0	11.2		*319*	4.0		*319*	7.2		*319*	14.9		*319*
Missing	*1*	0.0												
*Subjective social status*														
Low	*63*	2.8	**17.1**	*p⩽*0.001	*63*	**6.0**	*p⩽*0.001	*63*	**11.1**	*p⩽*0.001	*63*	**21.8**	*p⩽*0.001	*63*
Medium	*550*	24.1	**12.7**	*p⩽*0.001	*550*	**4.5**	*p⩽*0.001	*550*	**8.2**	*p⩽*0.001	*550*	**17.0**	*p⩽*0.001	*549*
High (ref)	*1666*	73.0	11.1		*1661*	4.0		*1662*	7.2		*1661*	14.9		*1661*
Missing	*4*	0.2												
Total	*2283*	100.0	11.7		*2277*	4.1		*2278*	7.5		*2277*	15.6		*2276*

Bold figures indicate a significant difference compared to the reference group (*p*⩽0.05).

Levels of self-reported stress followed the same pattern as SHCs; mean values were significantly higher among girls, adolescents living with one parent and those who live in families with low affluence or perceived their family’s social status as low or medium. Similar to SHCs, both objective and subjective socioeconomic conditions were important determinants of self-reported stress. Additionally, levels of stress were higher among adolescents with foreign background compared to those with Swedish background.

Logistic regressions analyses were performed to determine if the differences in SHC and self-reported stress with regards to gender, migration background, family structure and socioeconomic conditions shown in Table I remained when adjusting for all other variables. Adolescents reporting ‘at least two SHCs, always or often’ and the quartile of adolescents with the highest levels of stress were analysed. As results in Table II show, in the fully adjusted model girls had higher odds of reporting multiple SHCs and high stress than boys; adolescents living with one parent had higher odds of reporting high stress. Also, adolescents who perceived their families’ SSS as low or medium had higher odds of reporting multiple SHCs and high stress than those who perceived their families’ SSS as high. However, living in a low affluence family or having a migration background were no longer associated with levels of SHCs or stress.

**Table II.** Odds of reporting at least two subjective health complaints (SHCs) and high levels of self-reported stress among 13-year-olds, after gender, migration background, family structure and socioeconomic conditions, STARS 2015-2019.

	**At least two SHCs**				**High levels of stress**			
	Odds ratio	Lower 95% CI	Upper 95% CI	*p*	Odds ratio	Lower 95% CI	Upper 95% CI	*p*
*Gender*								
Boys	ref				ref			
Girls	**2.98**	2.45	3.62	*p*<0.001	**3.10**	2.45	3.93	*p*<0.001
*Migration background*								
Swedish background	ref				ref			
Foreign background	1.13	0.89	1.43	0.314	1.17	0.89	1.53	0.253
*Family structure*								
Both parents	ref				ref			
Joint custody	1.18	0.88	1.59	0.258	1.13	0.80	1.58	0.494
One parent	1.28	0.98	1.68	0.075	**1.36**	1.01	1.85	0.045
*Family affluence*								
Low	1.22	0.92	1.63	0.167	1.03	0.74	1.43	0.865
Medium	1.12	0.80	1.55	0.508	1.01	0.69	1.47	0.965
High	ref				ref			
*Subjective social status*								
Low	**4.54**	2.57	8.00	*p*<0.001	**6.26**	3.56	10.99	*p*<0.001
Medium	**1.82**	1.47	2.26	*p*<0.001	**1.71**	1.34	2.19	*p*<0.001
High	ref				ref			

Bold figures indicate a significant difference compared to the reference group (*p*⩽0.05).

### Associations between self-reported stress and SHCs

For the whole sample of students there was a strong association between self-reported stress and SHCs (*r*=0.70, Table III), meaning that 49 per cent of the variation in SHCs was explained by self-reported stress among adolescents. The association between self-reported stress and *somatic* health complaints was weaker than the association between self-reported stress and *psychological* complaints, *r*=0.52 compared to *r*=0.71 (*p*⩽0.001).

**Table III.** Linear correlation coefficients between self-reported stress and all health complaints, somatic complaints and psychological complaints, among 13-year-olds, by gender, migration background, family structure and socioeconomic conditions, STARS 2015-2019.

	Total complaints (0–32)			Somatic complaints (0–12)			Psychological complaints (0–20)		
	*r*	*n*	*p*	*r*	*n*	*p*	*r*	*n*	*p*
*Gender*									
Boys (ref)	0.65	*1009*		0.50	*1010*		0.65	*1009*	
Girls	0.69	*1266*	*0.057*	0.50	*1266*	*0.873*	**0.71**	*1266*	*0.011*
*Migration background*									
Swedish background (ref)	0.71	*1793*		0.52	*1793*		0.73	*1793*	
Foreign background	**0.66**	*475*	*0.049*	0.52	*476*	*0.849*	**0.65**	*475*	*0.004*
*Family structure*									
Both parents (ref)	0.69	*1686*		0.52	*1687*		0.70	*1686*	
Joint custody	**0.76**	*253*	*0.027*	0.54	*253*	*0.741*	**0.79**	*253*	*0.005*
One parent	0.63	*320*	*0.075*	0.49	*320*	*0.430*	0.64	*320*	*0.089*
*Family affluence*									
Low	0.69	*616*	*0.472*	0.53	*617*	*0.857*	0.70	*616*	*0.150*
Medium	0.69	*1340*	*0.465*	0.51	*1340*	*0.478*	0.70	*1340*	*0.190*
High (ref)	0.72	*319*		0.54	*319*		0.74	*319*	
*Subjective social status*									
Low	0.73	*63*	*0.522*	0.63	*63*	*0.204*	0.73	*63*	*0.569*
Medium	0.67	*549*	*0.497*	0.46	*549*	*0.136*	0.69	*549*	*0.905*
High (ref)	0.69	*1660*		0.52	*1661*		0.69	*1660*	
Total	0.70	*2275*		0.52	*2276*		0.71	*2275*	

Bold figures indicate statistically significant differences compared to the reference group (*p*⩽0.05) according to Fisher’s r-to-z transformation tests. *n* is the number of individuals in each group.

Overall, differences between subgroups were negligible when looking at associations between self-reported stress and total SHCs. There were however two exceptions: correlations were significantly weaker among adolescents with foreign background than among adolescents with Swedish background and stronger among those living in joint custody compared to adolescents living with both parents. No statistically significant differences between other subgroups in associations between self-reported stress and *somatic complaints* were found. Looking at associations between self-reported stress and *psychological complaints*, associations were significantly stronger among girls than boys, adolescents with Swedish background than among adolescents with foreign background and among adolescents living in joint custody compared to adolescents living with both parents. Multiple linear regression analyses were performed to determine whether the differences in associations found above remained when adjusting for possible confounders (gender, migration background and family structure). Results (not shown) showed that associations remained unchanged.

Finally, linear associations between self-reported stress and each of the eight SHCs are displayed in Table IV. Looking at the total sample of students, the strongest correlations were found between self-reported stress and felt sad (*r*=0.60), difficulties concentrating (*r*=0.55) and felt tense (*r*=0.53). The weakest correlations were found between self-reported stress and headache (*r*=0.38), stomach ache (*r*=0.44), felt dizzy (*r*=0.44) and difficulties in getting to sleep (*r*=0.44). With few exceptions, associations between self-reported stress and each of the eight health complaints did not differ between subgroups of adolescents.

**Table IV.** Linear correlation coefficients between self-reported stress and each subjective health complaint (SHC) among 13-year-olds, by gender, migration background, family structure and socioeconomic conditions, STARS 2015-2019.

	Concentrating difficulties		Sleeping difficulties		Headache		Stomach ache		Tense		Bad appetite		Sad		Dizzy	
	*r*	*n*	*r*	*n*	*r*	*n*	*r*	*n*	*r*	*n*	*r*	*n*	*r*	*n*	*r*	*n*
*Gender*																
Boys (ref)	0.50	*1009*	0.39	*1010*	0.36	*1010*	0.39	*1009*	0.47	*1009*	0.38	*1009*	0.55	*1010*	0.43	*1010*
Girls	0.55	*1266*	0.43	*1266*	0.36	*1266*	0.42	*1266*	**0.54**	*1266*	**0.45**	*1266*	0.58	*1265*	0.43	*1266*
*Country of birth*																
Swedish background (ref)	0.58	*1792*	0.44	*1793*	0.38	*1793*	0.44	*1792*	0.55	*1793*	0.46	*1793*	0.62	*1792*	0.45	*1793*
Foreign background	**0.46**	*476*	0.42	*476*	0.40	*476*	0.43	*476*	0.50	*475*	0.40	*475*	0.56	*476*	0.41	*476*
*Family structure*																
Both parents (ref)	0.55	*1686*	0.44	*1687*	0.39	*1687*	0.45	*1686*	0.53	*1686*	0.44	*1686*	0.58	*1686*	0.44	*1687*
Joint custody	0.55	*253*	0.43	*253*	0.37	*253*	0.41	*253*	0.58	*253*	**0.59**	*253*	0.63	*253*	0.50	*253*
One parent	0.48	*320*	0.35	*320*	0.33	*320*	0.42	*320*	0.46	*320*	0.37	*320*	0.58	*320*	0.41	*320*
*Family affluence*																
Low	0.52	*617*	0.47	*617*	0.39	*617*	0.48	*617*	0.52	*616*	0.43	*616*	0.59	*617*	0.40	*617*
Medium	0.54	*1339*	0.42	*1340*	0.38	*1340*	0.43	*1339*	0.55	*1340*	0.45	*1340*	0.59	*1339*	0.44	*1340*
High (ref)	0.61	*319*	0.41	*319*	0.38	*319*	0.42	*319*	0.49	*319*	0.49	*319*	0.61	*319*	0.51	*319*
*Subjective social status*																
Low	0.57	*63*	0.58	*63*	**0.60**	*63*	0.46	*63*	0.40	*63*	0.52	*63*	0.64	*63*	0.50	*63*
Medium	0.51	*549*	0.44	*549*	0.32	*549*	0.37	*549*	0.57	*549*	0.42	*549*	0.56	*548*	0.41	*549*
High (ref)	0.53	*1660*	0.40	*1661*	0.37	*1661*	0.45	*1660*	0.52	*1660*	0.44	*1660*	0.59	*1661*	0.42	*1661*
Total	0.55	*2275*	0.44	*2276*	0.38	*2276*	0.44	*2275*	0.53	*2275*	0.45	*2275*	0.60	*2275*	0.44	*2276*

Bold figures indicate statistically significant differences compared to the reference group (*p*⩽0.05) according to Fisher’s r-to-z transformation tests. *n* is the number of individuals in each group.

## Discussion

This study establishes that there are social inequalities in both SHCs and stress, attributable to gender, migration background, family structure and the family’s socioeconomic conditions. Results are in line with previous research regarding SHCs [5, 10] and the theoretical framework regarding stress from which we hypothesized that levels of stress would be influenced by social conditions (shown in Figure 1). We also found a social gradient in SHCs and stress when looking at the family’s subjective, but not objective, socioeconomic conditions.

This study also shows that self-reported stress and SHCs, especially psychological complaints, are strongly correlated among adolescents. The findings support the theoretical framework outlined in Figure 1 and are also in line with previous studies [20-23]. The findings implicate that SHCs, especially psychological complaints, among adolescents can be referred to as ‘stress-related’, and they further suggest that somatic complaints to a higher extent than psychological complaints may have other causes than stress, which is plausible as adolescence is a period of bodily changes and maturation.

In contrast to previous research linking stress to SHCs among adolescents, this study has taken the socioeconomic conditions of the family into account. Although the results showed that both SHCs and stress are more prevalent among adolescents in low affluence and low or medium subjective social status families, the association between stress and SHCs did not vary with socioeconomic conditions. In other words, socioeconomic conditions did not influence the associations. Gender, migration background and family structure influenced the associations between stress and health complaints to some extent. Consequently, we found some support for our hypothesis (Figure 1) that the association between stress and SHCs would be influenced by social conditions.

## Conclusions

It is plausible that the increase of mental ill health seen among adolescents in Sweden, as well as in many other countries, are a result of increased stress in society. Consequently, measures aimed at improving Swedish adolescents’ mental health by reducing levels of SHCs should pay special attention to adolescents’ appraisal and coping of stressors in their daily lives, such as their family context and working conditions in school [17].

The results show that psychological complaints are more common and have a stronger association to stress among adolescents living in joint custody. As joint custody has become increasingly common in Sweden, we recommend future research on the importance of living in joint custody for stress and SHCs.

### Strengths and limitations

Broad definitions, together with validated composite measurements, of both stress and SHCs have been applied in this study. Further, the sample is relatively large compared to other similar studies (e.g. [21-23] where samples range from 1027 to 1233 students). Although STARS is based on a regional sample and the response rate was 45 per cent, the sample is comparable to official statistics and the Swedish HBSC study with regards to migration background and family structure. Further, family affluence and the prevalence of recurrent headache, stomach ache and dizziness is similar to that among 13-year-olds in the HBSC 2017/18 [13]. Therefore, we believe that the results are valid for the Swedish adolescent population as a whole.

Using cross-sectional data means that we cannot confirm the direction of the correlations. Although our theoretical framework states that stress is an underlying mechanism of SHCs, the opposite is possible; experiencing several recurrent SHCs may cause stress among children and adolescents as it may impair their school attendance and school performance, social relationships and leisure activities.

There is a risk of recall bias for the retrospective self-reports on SHCs (during the last 6 months) and perceived stress (during the last month). The strong association between the two implicates that the students’ responses actually reflect their current health status or health status over the last couple of weeks. This is supported by validation work of the PSS [25] showing high test–re-test reliability after 2 days, but not after 6 weeks. Also, validation work of the HBSC-SCL showed high test–re-test variability after 1 week [30].

## References

[1] Bor W Dean AJ Najman J , et al. Are child and adolescent mental health problems increasing in the 21st century? A systematic review. Aust N Z J Psychiatry 2014;48:606-16. 24829198 10.1177/0004867414533834

[2] Collishaw S. Annual research review: Secular trends in child and adolescent mental health. J Child Psychol Psychiatry 2015;56:370-93. 25496340 10.1111/jcpp.12372

[3] Gustafsson J-E Allodi Westling M Åkerman A , et al. School, learning and mental health: a systematic review. Kungliga vetenskapsakademien; 2010.

[4] National Board of Health and Welfare [Socialstyrelsen]. Psykisk ohälsa bland unga. Underlagsrapport till Barns och ungas hälsa, vård och omsorg 2013. Stockholm; 2013.

[5] Cavallo F Zambon A Borraccino A , et al. Girls growing through adolescence have a higher risk of poor health. Qual Life Res 2006;15:1577-85. 17033911 10.1007/s11136-006-0037-5

[6] Ravens-Sieberer U Torsheim T Hetland J , et al. Subjective health, symptom load and quality of life of children and adolescents in Europe. Int J Public Health 2009;54 Suppl 2:151-9. 19639258 10.1007/s00038-009-5406-8

[7] Inchley J Currie D Budisavljevic S , et al. Spotlight on adolescent health and well-being. Findings from the 2017/2018 Health Behaviour in School-aged Children (HBSC) survey in Europe and Canada. International report. Volume 1. Key findings. Copenhagen; 2020.

[8] Hagquist C. Psychosomatic health problems among adolescents in Sweden—are the time trends gender related? Eur J Public Health 2009;19:331-6. 19304732 10.1093/eurpub/ckp031

[9] Friberg P Hagquist C Osika W. Self-perceived psychosomatic health in Swedish children, adolescents and young adults: an internet-based survey over time. BMJ Open 2012;2:e000681. 10.1136/bmjopen-2011-000681 PMC4400616 22855621

[10] Ottova V Erhart M Vollebergh W , et al. The role of individual- and macro-level social determinants on young adolescents’ psychosomatic complaints. J Early Adolesc 2012;32:126-58.

[11] Ahlborg M Svedberg P Nyholm M , et al. Socioeconomic inequalities in health among Swedish adolescents: adding the subjective perspective. BMC Public Health 2017;17:838. 29061173 10.1186/s12889-017-4863-x PMC5653986

[12] Svedberg P Nygren JM Staland-Nyman C , et al. The validity of socioeconomic status measures among adolescents based on self-reported information about parents’ occupations, FAS and perceived SES; implication for health related quality of life studies. BMC Med Res Methodol 2016;16:48. 27130331 10.1186/s12874-016-0148-9 PMC4850630

[13] Public Health Agency of Sweden [Folkhälsomyndigheten]. Skolbarns hälsovanor i Sverige 2017/18 – grundrapport. Stockholm; 2019.

[14] National Board of Health and Welfare [Socialstyrelsen]. Utvecklingen av psykisk ohälsa bland barn och unga vuxna till och med 2016. Stockholm; 2017.

[15] Public Health Agency of Sweden [Folkhälsomyndigheten]. Varför har den psykiska ohälsan ökat bland barn och unga i Sverige? Utvecklingen under perioden 1985-2014. Stockholm; 2018.

[16] Anniko M. Stuck on repeat Adolescent stress and the role of repetitive negative thinking and cognitive avoidance. PhD thesis, Örebro University, 2018.

[17] Ostberg V Plenty S Laftman SB , et al. School demands and coping resources-associations with multiple measures of stress in mid-adolescent girls and boys. Int J Environ Res Public Health 2018;15:2143. 10.3390/ijerph15102143 PMC6209916 30274260

[18] Lazarus RS Folkman S. Stress, appraisal, and coping. New York: Springer, 1984.

[19] Pearlin L Lieberman M Menaghan E , et al. The stress process. J Health Soc Behav 1981;22:337-56. 7320473

[20] Sundblad GB Jansson A Saartok T , et al. Self-rated pain and perceived health in relation to stress and physical activity among school-students: a 3-year follow-up. Pain 2008;136:239-49. 17709208 10.1016/j.pain.2007.06.032

[21] Wiklund M Malmgren-Olsson EB Ohman A , et al. Subjective health complaints in older adolescents are related to perceived stress, anxiety and gender: a cross-sectional school study in Northern Sweden. BMC Public Health 2012;12:993. 23158724 10.1186/1471-2458-12-993 PMC3533931

[22] Moksnes UK Lazarewicz M. The association between stress, resilience, and emotional symptoms in Norwegian adolescents from 13 to 18 years old. J Health Psychol 2019;24:1093-102. 28810383 10.1177/1359105316687630

[23] Moksnes UK Espnes GA. Sense of coherence in association with stress experience and health in adolescents. Int J Environ Res Public Health 2020;17:3003. 10.3390/ijerph17093003 PMC7246660 32357461

[24] Hagquist C. Psychometric properties of the psychosomatic problems scale: a Rasch analysis on adolescent data. Soc Indic Res 2008;86:511-23.

[25] Cohen S Kamarck T Mermelstein R. A global measure of perceived stress. J Health Soc Behav 1983;24:385-96. 6668417

[26] Torsheim T Cavallo F Levin KA , et al. Psychometric validation of the revised family affluence scale: a latent variable approach. Child Indic Res 2016;9:771-84. 27489572 10.1007/s12187-015-9339-x PMC4958120

[27] Inchley J Currie D Young T , et al. Growing up unequal: gender and socioeconomic differences in young people’s health and well-being. Health Behaviour in School-aged Children (HBSC) Study: International Report from the 2013/14 Survey. Copenhagen; 2016.

[28] Inchley J Currie D Cosma A , et al. Health Behaviour in School-aged Children (HBSC) Study Protocol: background, methodology and mandatory items for the 2017/18 survey. St Andrews; 2018.

[29] Goodman E Adler NE Kawachi I , et al. Adolescents’ perceptions of social status: development and evaluation of a new indicator. Pediatrics 2001;108:E31. 11483841 10.1542/peds.108.2.e31

[30] Hetland J Torsheim T Aarø L. Subjective health complaints in adolescence: dimensional structure and variation across gender and age. Scand J Public Health 2002;30:223-30. 12227979 10.1080/140349402320290953

